# Hierarchically patterned self-powered sensors for multifunctional tactile sensing

**DOI:** 10.1126/sciadv.abb9083

**Published:** 2020-08-19

**Authors:** Yang Wang, Heting Wu, Lin Xu, Hainan Zhang, Ya Yang, Zhong Lin Wang

**Affiliations:** 1 CAS Center for Excellence in Nanoscience, Beijing Key Laboratory of Micro-nano Energy and Sensor, Beijing Institute of Nanoenergy and Nanosystems, Chinese Academy of Sciences, Beijing 100083, China.; 2 School of Nanoscience and Technology, University of Chinese Academy of Sciences, Beijing 100049, China.; 3 Center on Nanoenergy Research, School of Physical Science and Technology, Guangxi University, Nanning 530004, China.; 4 School of Material Science and Engineering, Georgia Institute of Technology, Atlanta, GA 30332-0245, USA.

## Abstract

Flexible sensors are highly desirable for tactile sensing and wearable devices. Previous researches of smart elements have focused on flexible pressure or temperature sensors. However, realizing material identification remains a challenge. Here, we report a multifunctional sensor composed of hydrophobic films and graphene/polydimethylsiloxane sponges. By engineering and optimizing sponges, the fabricated sensor exhibits a high-pressure sensitivity of >15.22 per kilopascal, a fast response time of <74 millisecond, and a high stability over >3000 cycles. In the case of temperature stimulus, the sensor exhibits a temperature-sensing resolution of 1 kelvin via the thermoelectric effect. The sensor can generate output voltage signals after physical contact with different flat materials based on contact-induced electrification. The corresponding signals can be, in turn, used to infer material properties. This multifunctional sensor is excellent in its low cost and material identification, which provides a design concept for meeting the challenges in functional electronics.

## Teaser

A multifunctional self-powered sensor is developed for pressure, temperature, and material sensing.

## INTRODUCTION

Humans can apperceive pressure and temperature and deduce their material properties while applying contact between objects and skin (*1*). The development in functional electronics reveals strategies that allow us to realize some tactile functions of human skin (*2*–*6*). For example, electronic skins and flexible sensors have been implemented in robotics and wearable health-monitoring devices to detect ambient changes of strain, vibration, and the direction of applied pressure (*7*–*13*). In addition, graphene channels were exploited to render stretchable thermistor (*14*), and stretchable-gated sensors were used to gather the temperature of the object (*15*). Recently, even more fascinating sensors can simultaneously detect pressure and thermal variations in a single device using ferroelectric or organic thermoelectric materials (*16*–*18*). An important future to imitate the feature of human skin lies in the development of multifunctional sensing, especially for inferring material properties.

Inferring material properties of objects is needed for many industrial and medical conditions (*19*, *20*). A number of technologies, including various image pattern recognition methods and machine learning technology, have been implemented in material identification (*21*–*22*). For example, Sundaram *et al.* (*23*) recently demonstrated a scalable tactile glove that can perceive individual objects by deep convolution neural networks. Although these approaches have promoted the performance boundaries for identifying objects, their potential to infer smooth materials and further improve their capability may be limited by the use of grasping signals and complex algorithms. It is advantageous for the systems to directly use signals generated from sensors without the complicated data processing.

Triboelectric nanogenerator (TENG) is a promising alternative approach to bridging the technological gap (*24*, *25*). Recently, the TENG based on the coupling of triboelectrification and electrostatic induction has been investigated for energy harvesting and self-powered mechanical sensing (*26*–*29*). For example, Guo *et al.* (*30*) demonstrated a triboelectric auditory sensor for both robotics and human beings. Triboelectrification between different materials can indicate the natural physical property of materials; however, it mainly suffers from the applied pressure and temperature. Integrating a TENG with other sensors may provide a simple solution to realize skin-like sensing systems.

Here, we present a multifunctional, tactile self-powered sensor that enables pressure, temperature, and material sensing. The constitution takes the form of a multilayer stack: (i) a hydrophobic polytetrafluoroethylene (PTFE) film as the electrification layer, (ii) two Cu sheets coated with the silver nanowires (Ag NWs) film as electrodes, and (iii) a sponge-like graphene/polydimethylsiloxane (PDMS) composite as the responsive component to piezoresistive and thermoelectric effects. The device characterizes with a high-temperature detection resolution and a pressure-sensing sensitivity of 1 K and 15.22 kPa^−1^, respectively. The key concept of our device lies in inferring material properties based on generated electric signals between the PTFE film and objects. We introduce a simple algorithm—lookup table algorithm operated with MATLAB—to analyze the signals on the computer. As proof of concept, we indicate that the device can infer 10 different flat materials. This work opens up new paths for using self-powered sensors in multifunctional tactile sensing.

## RESULTS

### Materials, mechanism, and design strategies

**Fig. 1:**
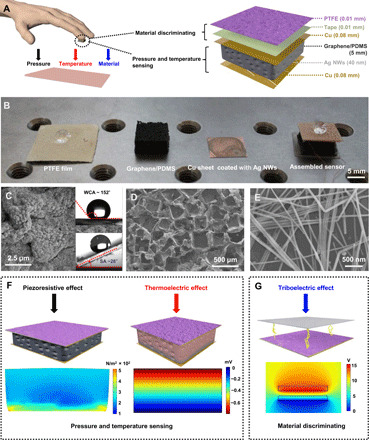
Structure and working mechanism of the multifunctional sensor. (**A**) The sensor can be attached to a human finger, allowing multifunctional tactile sensing. Exploded-view schematic diagram shows that the sensor takes the form of a multilayer stack. (**B**) Photograph showing main components and the assembled sensor. (**C**) The SEM image of the prepared hydrophobic PTFE surface. (**D**) The SEM image of the prepared graphene/PDMS composite surface. (**E**) The SEM image of synthesized Ag NWs. (**F**) The simulated strain field (left) and electric potential (right) on the graphene/PDMS composite when the composites are subjected to the applied pressure and temperature gradient, respectively. (**G**) The electric potential on the PTFE when it is in contact with the object. Photo credit: Ya Yang, Beijing Institute of Nanoenergy and Nanosystems.

Figure 1 (A and B) shows the schematic illustration and the optical image of the multifunctional tactile sensor that mainly consists of a hydrophobic PTFE film, a double-sided tape, a graphene/PDMS sponge, and two Cu sheets (the fabrication process is described in Materials and Methods and fig. S1). Our sensor design comprises two vertically stacked active parts to achieve the independent discrimination of pressure, temperature, and material properties. The PTFE used as the electrification layer, which will contact the surface of perceptive objects, is located on the top Cu sheet via the tape. Compared with other materials, such as polyurethane, nylon (polyamide), and polyethylene terephthalate, the PTFE has advantages of durability and water repellency. To enhance the surface charge density and hydrophobicity, we created micro/nanoporosities on the PTFE film by mixing zinc acetate (ZnAc_2_) and sodium chloride (NaCl) into the PTFE emulsion. The scanning electron microscopy (SEM) image (Fig. 1C) shows the dual-scale nature of the porosities. The water contact angle (WCA) at its upper right corner shows 152°, and the sliding angle (SA) at its lower right corner exhibits 28°. X-ray diffraction (XRD) results (fig. S2A) indicate the expected patterns for PTFE and ZnO. Using thermoelectric material and flexible frame, namely, graphene and PDMS, we created the deformable microstructure sponge responded to varied pressure and temperature via the simple mixed approach. The choice of porous composites is essential to realize the desired electrical conductivity and thermal insulation. As shown in the SEM image (Fig. 1D), the composites feature interconnected pores with an average pore size of ~200 μm. To achieve the desired conductivity and sensing characteristics, we deposited Ag NWs onto the surface of Cu sheets by simple dip-coating and drying. When the solution was completely evaporated, the residual polyvinyl pyrrolidone (PVP) can connect the Ag NWs to the Cu sheet. After 10 stress tests, the Ag NWs film remains relatively intact, showing favorable adhesion between the Ag NWs and the sheet (fig. S2, C and D). The SEM image (Fig. 1E) demonstrates the uniform deposition of Ag NWs with a diameter of ~110 nm, and the XRD patterns (fig. S2B) show the corresponding phase. The entire assembly of the device is a simple process incorporating lamination and packaging with an ultraviolet (UV)–cured glue.

The multifunctional sensing of our sensor suggests that it might operate via different mechanisms. First, the sensing mechanism for pressure is due to piezoresistive sensing, which uses the contact resistance between the electrode and the conducting graphene/PDMS sponge. The mechanics for the force-dependent contact are apparent from the finite element analysis results of Fig. 1F (left). According to the thermoelectricity, our device shows the temperature-sensing properties when it contacts with an object with temperature stimuli. The stimuli can induce the voltage (*V*_therm_), which is defined as *V*_therm_ = *S*_T_ × Δ*T*, where *S*_T_ represents the Seebeck coefficient and Δ*T* represents the temperature gradient of the sensor (Fig. 1F, right). To realize material identification, we took the advantage of triboelectrification, a well-known phenomenon that exhibits the natural physical property of materials. When an object contacts with the PTFE film, electricity transport appears due to Maxwell’s displacement current (Fig. 1G).

### Piezoresistance property of the device

**Fig. 2:**
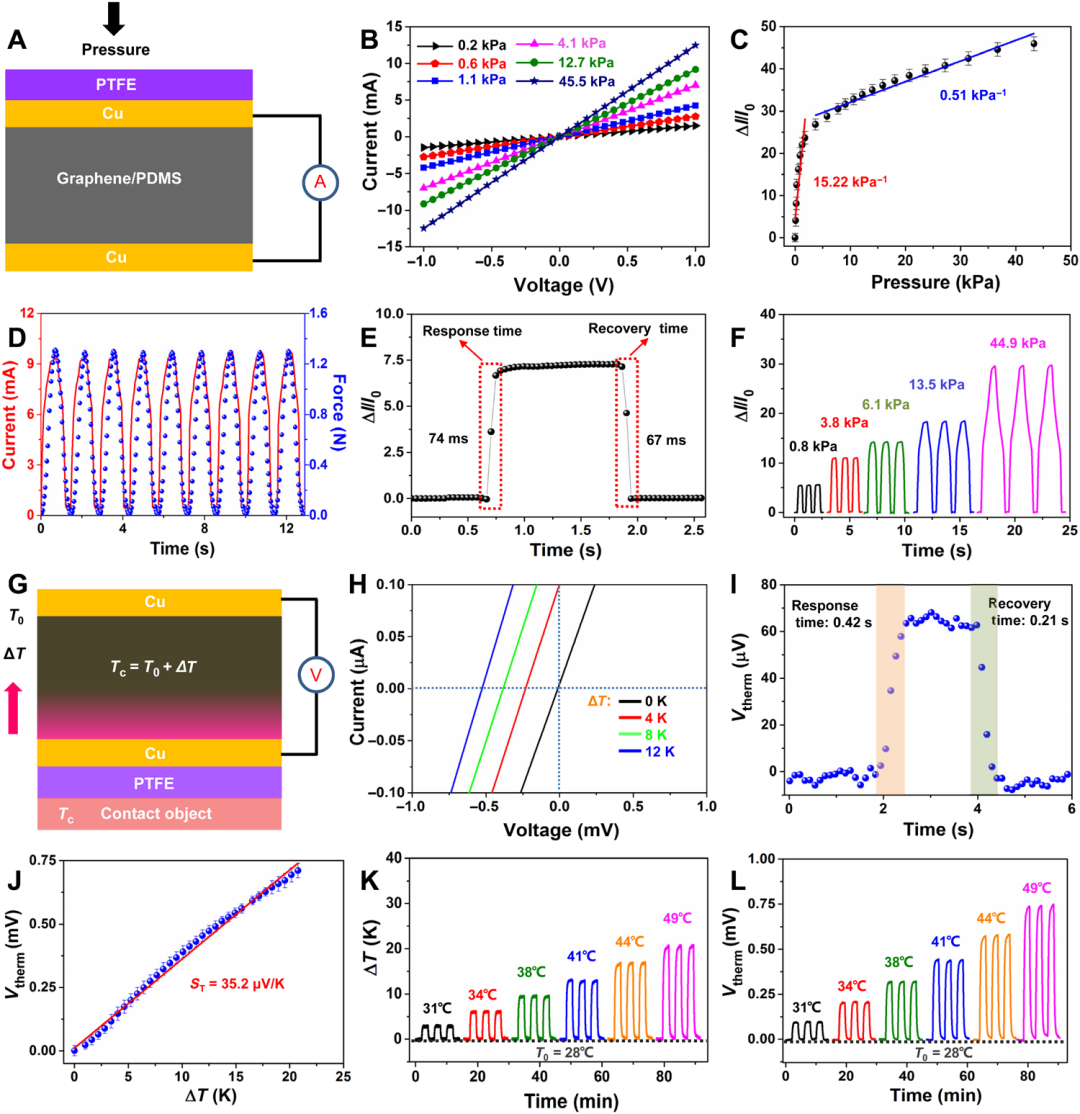
Electric characterization of the sensor in response to pressure and temperature. (**A**) Schematic illustrations of the pressure and the measured mode of electrical signals. (**B**) The linear relation of the *I-V* curves illustrates the ohmic contacts between graphene/PDMS composite and Cu sheet electrodes. (**C**) The Δ*I*/*I*_off_ first exhibits a sharp increase as function of a pressure below 4 kPa and then a small increase above 4 kPa. Error bars were calculated by five sets of data under each pressure. (**D**) Both the output current and external pressure on time kept well in step with the loading and unloading. (**E**) *I-T* curve exhibiting fast responsive and recovery times of <80 ms. (**F**) The current monotonically increases with the pressure. (**G**) Schematic illustrations of the temperature gradient and the measured mode of electrical signals. *T*_0_ is the ambient temperature, and *T*_c_ is the temperature of the object. (**H**) The linear relation of the *I-V* curves illustrates the constant shifting at various Δ*T*. (**I**) *V-T* curve exhibiting fast responsive and recovery times of <0.5 s. (**J**) Measured output voltage as a function of temperature gradient. (**K**) Measured temperature gradient curves of the graphene/PDMS composite. (**L**) Measured output voltage signals corresponding to the temperature gradient.

To demonstrate the pressing responses of the sensor, we performed the current measurement under various levels of pressure (Fig. 2A). The typical linear relationship of the current-voltage (*I-V*) (Fig. 2B) curves indicates that the favorable ohmic contacts formed between the graphene/PDMS sponge and electrodes. The pressure sensitivity of our sensor is defined as *S* = (Δ*I*/*I*_0_)/Δ*P*, where Δ*I* is the corresponding change in current, *I*_0_ is the initial current of the device without pressure loading, and Δ*P* is the changing pressure. As shown in Fig. 2C, the sensitivity below the pressure of 5 kPa shows a higher value of 15.22 kPa^−1^. In the high-pressure range (5 to 45 kPa), the sensitivity dropped to 0.51 kPa^−1^. The contact between Ag NWs and the graphene/PDMS sponge led to more conductive pathways under the small pressure (*31*). They played a prominent role in promoting pressure sensitivity and better electrical and physical contacts. We investigated the sensitivity of the device without Ag NWs and found a lower value of 0.32 kPa^−1^ (fig. S3A). In addition, the NaCl and graphite mass ratio also affected the sensitivity, because the optimized design could form a favorable balance between the conductivity and the deformation of the composite sponge (fig. S3, B and C). For the thickness of the graphene/PDMS sponge, higher sensitivity was obtained under a larger thickness in the pressure range from 5 to 40 kPa (fig. S3D). However, the graphene/PDMS with a thickness of about 10 mm is a little bulky. Considering that the sensors might be used for wearable sensing, we used the graphene/PDMS with a thickness of 5 mm as the responsive component. Figure S3E, showing the mechanical responses of graphene/PDMS sponges, demonstrates that the maximum strain and elastic modulus are about 70% and 16.2 kPa, respectively.

**Fig. 3:**
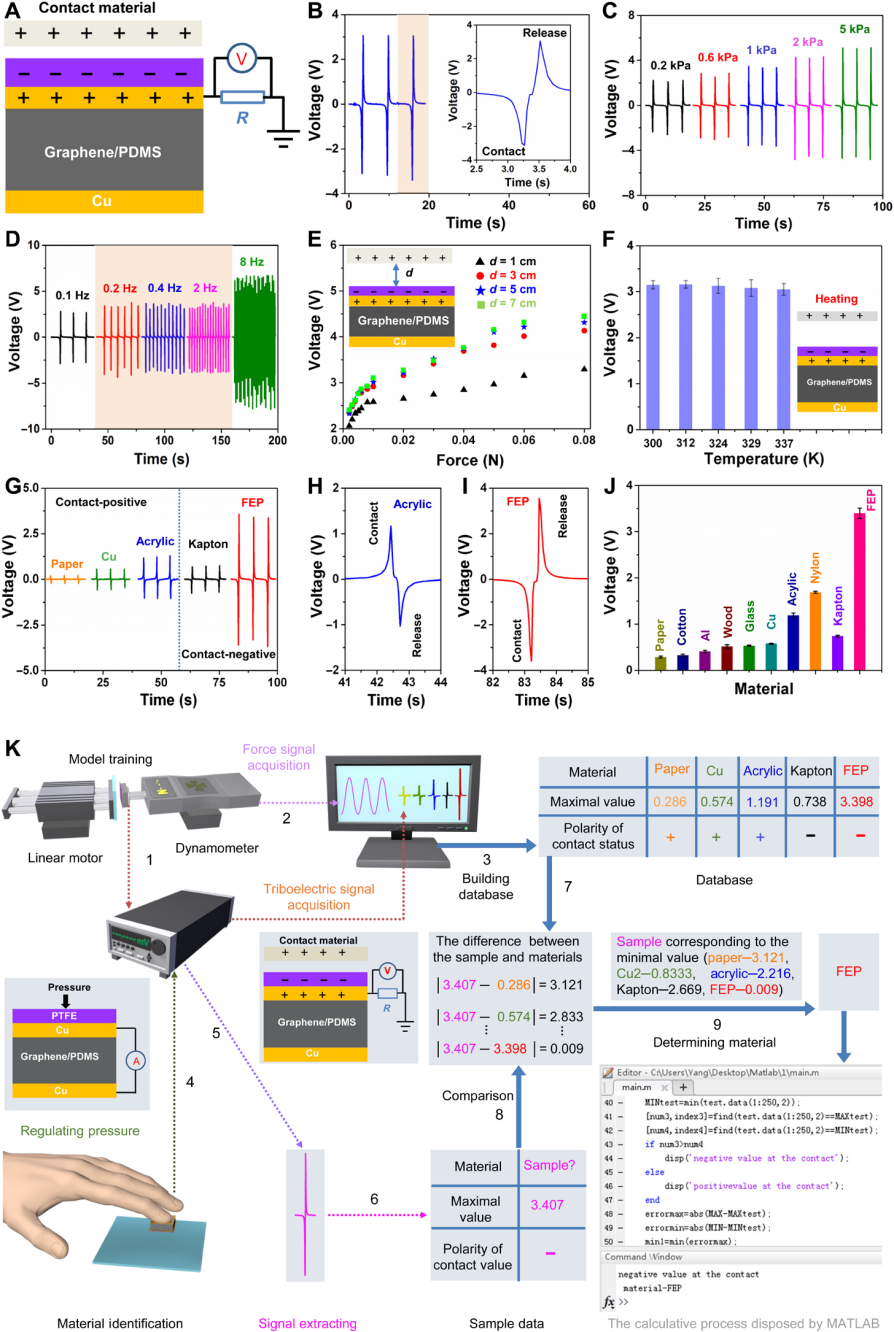
Characterizing the performance of the TENG and identifying materials of objects from tactile-induced triboelectric signals. (**A**) Schematic illustrations of the TENG and the measured mode of electrical signals. (**B**) Output voltage signals of TENG for FEP (*R* = 100 megohms, *d* = 3 cm, frequency = 0.2 Hz). (**C**) Output voltage signals of TENG under different pressure values. (**D**) Output voltage signals of TENG illustrating the same values between 0.2 and 2 Hz. (**E**) The influence of gap distance for the TENG. (**F**) The influence of temperature for the TENG. (**G**) Output voltage signals of the TENG for five materials. (**H**) Enlarged curve of the signal for acrylic. (**I**) Enlarged curve of the signal for FEP. (**J**) Summarized peak amplitude. Data are means ± SEM. Error bars were calculated by five sets of data. (**K**) The two-factor material identification system. The system consists of the training process (1 to 3) and the identification process (4 to 9).

Below, we discuss the response time and stability of our device to external forces. The output electrical signals were almost the same as the input pressure waves under a pressure of about 1.3 N, which demonstrates a negligible hysteresis (Fig. 2D). Regarding the frequency response, the output signals were stable without obvious change (fig. S3F). Our sensor showed an instant response to both external loading and unloading, as illustrated in Fig. 2E. The response and recovery times faster than 80 ms keep the sensor sensing in time with the mechanical stimuli. By increasing the pressure value, the electric current gradually increased, demonstrating stable and continuous waves (Fig. 2F). The minimum detectable pressure of the sensor was measured by loading/unloading the small force of 0.01 N, as shown in fig. S3G. Electrical signals of the sensor were steady after 3000 cycles in a loading-unloading test (Fig. 3H). In addition, the temperature stimuli exhibit a negligible effect on the current change of the devices (fig. S3I). These pressure-sensing performances of the device can meet the requirements of many practical applications.

### Self-powered temperature sensing of the device

To investigate the responses of our sensors to temperature, we built a homemade system. It mainly contains a semiconductor thermoelectric cooler sheet as the heating source and the infrared thermal imaging to record temperature variations (fig. S4A). Note that we measured temperature gradient–induced voltage signals, because current signals suffered from the applied temperature (Fig. 2G). The PTFE films used as the electrification layer should be in contact with objects to realize material identification. However, the use of PTFE films will reduce *V*_therm_ responses of devices (fig. S4B). Figure 2H illustrates the constant shifting of the *I-V* curve with increasing the temperature gradient. Our sensor exhibited an immediate response and a resolution to a temperature signal. The voltage of about 60 μV was measured under a small Δ*T* of 1 K, demonstrating a precise temperature resolution of the sensor, as shown in Fig. 2I. It also shows response and recovery times of 0.42 and 0.21 s, respectively. Figure 2J shows the voltage (*V*_therm_) as a function of the temperature gradient from 0 to 22 K, with a moderate *S*_T_ of 35.2 μV/K. These results illustrate that the stable thermoelectric conversion permits sensitive detection for temperature variations.

**Fig. 4:**
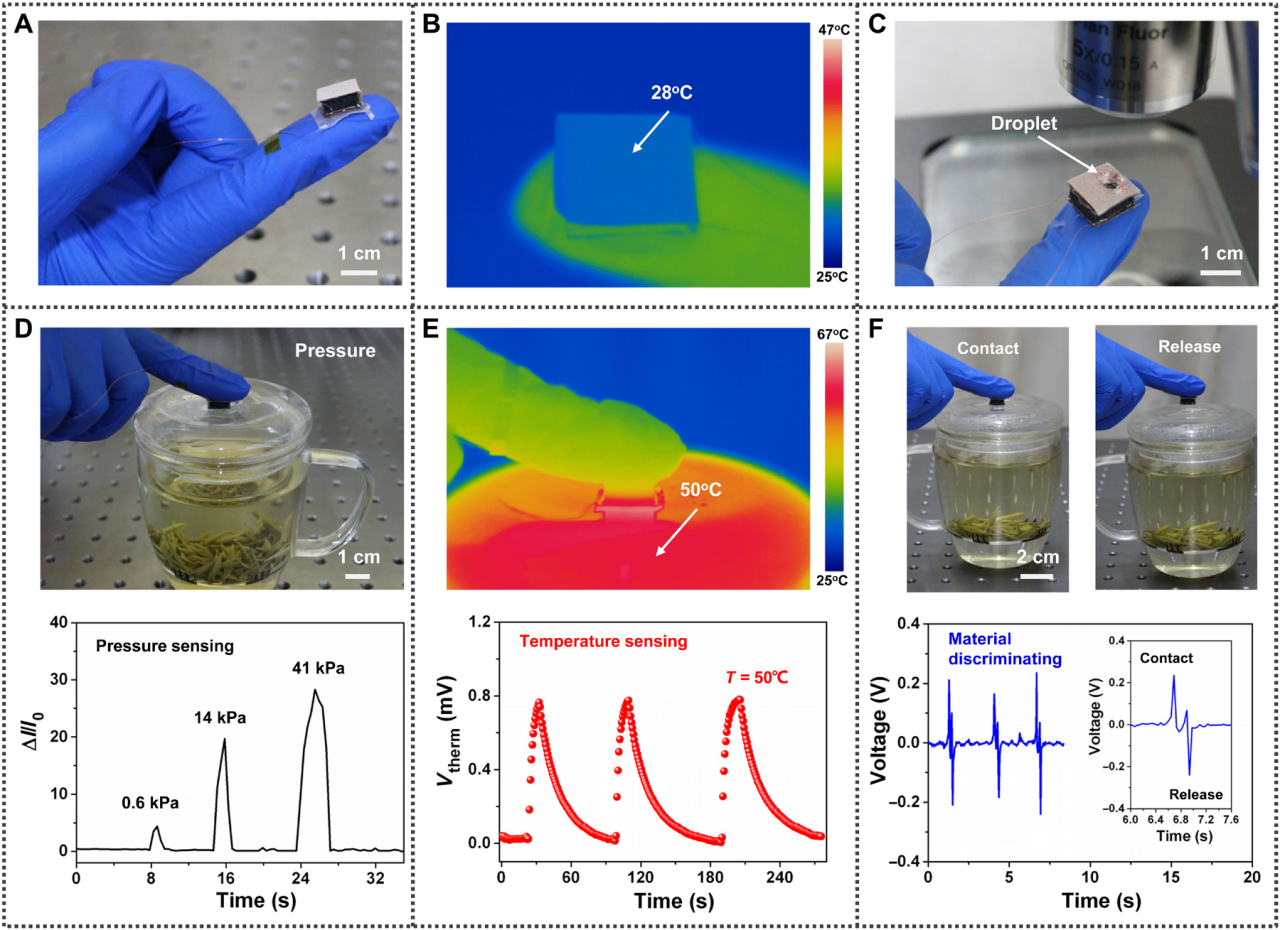
Examples for the applications of sensors. (**A**) The sensor on a human finger showing the potential applications in wearable devices. (**B**) The infrared thermal image showing the temperature of the sensor. (**C**) Optical image showing the sensor controlling a water droplet for biomedical applications. (**D**) An optical image of a finger with the sensor touching a hot cup and a plot for the corresponding current change of the sensor. (**E**) Thermal image of a finger with the sensor touching a hot cup and a plot for the *V*_therm_ responses of the sensor. (**F**) Images showing operations of the finger to contact with the cup and release. Plots show contact-induced electrification responses to a contact-release movement. Photo credit: Ya Yang, Beijing Institute of Nanoenergy and Nanosystems.

Considering the stable temperature-sensing response, we investigated the temperature gradient and the gradient-induced voltage signals to various levels of temperature. It should be mentioned that the pressure signal shows a limited effect on *V*_therm_ (fig. S4C). Figure 2K, showing the temperature difference curves, demonstrates that the fabricated graphene/PDMS sponge with the favorable adiabatic property is important to use the thermoelectric effect. Corresponding to the temperature difference ranging from 4 to 22 K, the measured voltage signals gradually increase, as shown in Fig. 2L. The thickness of the graphene/PDMS sponge also demonstrates an effect on *V*_therm_ (Fig. 4D). Our sensors with outstanding thermal stability can perform well under different conditions, making it adaptable for intelligent wearable elements to probe hot objects.

### Fundamental characteristics of TENG

We systematically discussed the fabricated TENG, which manipulates in a single-electrode mode using the top Cu sheet as the electrode (Fig. 3A). Because of its stability and the wide range of applications, the fluorinated ethylene propylene (FEP) membrane was chosen as the contacting material for investing the performance of TENG. When the membrane is in contact with the top PTFE film, a transfer of surface charges appears due to different abilities to electron affinities (fig. S5). By increasing the separation distance between the two membranes, the induced positive charges form on the electrode, leading to an output voltage signal to a load. When the two membranes approach again, a reversed output signal appears. Figure 3B shows the obtained voltage under a load of 100 megohms with a contact area of 1 cm by 1 cm. The inset illustrates an enlarged view of generated voltage in one cycle, indicating that the output signal has two opposite peak values corresponding to different statuses. This result illustrates that the output signals generated by TENG enable sensitive detection for the contacting material.

To understand the performance of the TENG, we built a simple system consisting of a computer-controlled linear motor and a dynamometer. The FEP membrane was fixed on the motor to implement pressure uniformly onto the sensor. Meanwhile, the dynamometer can record the pressure on the sensor. Figure 3C, illustrating the *V-T* curves with the pressure below 5 kPa under the laboratory environment, demonstrates that the output voltage increases with the applied pressure. Each curve has two opposite peak values under each contact and release. Our TENG probes a minimal pressure of 0.2 kPa, with an output voltage of about 2.5 V. In Fig. 3D, a series of *V-T* curves under different frequencies were measured. The TENG exhibited enhanced voltage when operating at a high frequency of 8 Hz. Note that the output voltage of TENG, corresponding to the difference ranging from 0.2 to 2 Hz, is steady and continuous, enabling their hopeful applications in ingestible electronics.

Furthermore, we investigated the influence of the detached distance and the temperature of the FEP membrane. As shown in Fig. 3E, comparable voltage responses to the varied pressure were observed when the distance was retained at 1 to 7 cm. The TENG exhibited high pressure–sensing sensitivities in the low (<0.01 N) pressure. The measured voltage signals were almost the same under the corresponding pressure when the distance was relatively large (>3 cm). These results are consistent with recent reports (*30*). Regarding the temperature effect, we investigated the voltage signals to various temperatures. Because of the TENG mechanism, the output voltage exhibited a slight dependence on the temperature of objects at a specific temperature range (Fig. 3F). These properties demonstrate that the applied pressure, regardless of the temperature variation and the detached distance, played an important role in affecting the output voltage.

### Self-powered material identification of the device

The uniqueness of our sensors is their ability to realize material identification. Any two different materials can induce triboelectric signals due to universal contact electrification (*32*). On the basis of this property, our devices exhibited diverse output signals when the PTFE film contacted with other materials. First, to ensure the distinct description of this process, we investigated five flat materials that were fixed on the linear motor to keep the identical pressure and frequency (Fig. 3K). Figure 3G, showing the *V-T* curves with different materials, demonstrates that the natural property of materials affects the polarity and the value of the output voltage. Paper, Cu, and acrylic are tending to lose electrons when they are in contact with the PTFE film, leading to a positive voltage signal (Fig. 3H). In contrast, Kapton and FEP are more tribo-negative, with a negative voltage signal (Fig. 3I). In addition, the property of materials decided the amplitude of output voltage due to their inherent ability of losing/gaining electrons. Results in Fig. 3J show that the obtained output voltage of FEP is higher than that of other materials. The distinction of the output value and the polarity generated by PTFE film and contacting materials makes it applicable for inferring material properties.

Humans can infer material properties while applying the suitable force on the object. Motivated by this, we propose a simple strategy of material identification (Fig. 3K). We first performed a model training process, in which the contacting force and output voltage signals were measured by the dynamometer and the digital source meter, respectively (Fig. 3K, 1 and 2). The output voltage tests depicted above help to assess the capability of our device in capturing useful data. The device can be attached to a human or robotic finger to infer the property of materials. The ability to estimate the contacting force is important for inferring material properties. Here, we used the piezoresistive property of the device to apply the right amount of force (Fig. 3K, 4). Next, the output voltage of the testing object was investigated by the TENG. To avoid influence between the different objects, UV irradiation can be used to remove surface charges on the PTFE film after each test (*33*).

To infer material properties, we developed a simple algorithm operated with MATLAB, namely, the lookup table algorithm. It can process the voltage signals from the sensors. First, the voltage signals of five materials were converted to a database, which consisted of maximal voltage values and the polarity (Fig. 3K, 3). When the minimum absolute value of those materials is larger than the acceptable tolerance (0.02 V), the property of testing materials can be obtained (Fig. 3K, 7 to 9). Here, we observed that the accuracy of identifying the FEP is high because of its outstanding electrification (table S1). In addition, the accuracy slightly reduced when the range of investigating materials was enlarged (table S2). Note that our devices can operate favorably on many practical applications, and our strategy is simple compared with conventional machine learning technology.

### Applications of the sensor for wearable device

We attached the sensor, serving as a wearable electronic device, lightly to a human finger via double-sided adhesive tape to illustrate its potential applications (Fig. 4A). The infrared thermal image shows that the surface temperature of the sensor is lower than that of the finger (Fig. 4B). Recently, biological systems with special wettability have been developed to overcome many academic and industrial problems (*34*, *35*). Our sensors with the hydrophobic property would make human or robotic fingers more applicable to control liquid. As shown in Fig. 4C, we can conveniently control a small water drop and observe it by the optical microscope. This liquid-controlled performance of the device can meet the requirements of many biological and biomedical applications.

Perceiving the temperature of the contacting object and inferring their material properties could enable humans or robots to interact with the real world. Next, we demonstrated the use of the sensor for realizing temperature sensing and material identification. To investigate the pressure- and temperature-sensing performances of the device, we monitored current changes and the obtained *V*_therm_ when touching a cup of hot drink, respectively. The pressure sensor showed rapid responses and recognized the difference in applied pressure on the cup, as illustrated in Fig. 4D (movie S1). It could help robots or disabled patients to apply the right amount of force on objects. Temperature sensing is another essential function, which can provide information about the temperature of contacted objects. The temperature sensor demonstrated a *V*_therm_ response to the cup with a temperature of 50°C (Fig. 4E and movie S2). Our devices exhibit an outstanding functionality that can realize the material identification based on universal contact electrification. The TENG can generate output voltage signals in response to the contact-release movement (Fig. 4F and movie S3). Integrating these voltage signals with the lookup table algorithm could help robots to realize the material identification of contacted objects. Moreover, our device can enable pressure, temperature, and material sensing for flexible objects (fig. S6).

## DISCUSSION

We present material and structural designs that enable a multifunctional sensing mechanism. Incorporating the graphene into the PDMS via a simple template method, sponge-like conducting composites have been achieved. The composite not only has outstanding electrical properties but also exhibits thermoelectric features. This capability makes it a prospective element for temperature-pressure sensing. In addition, we prepared hydrophobic PTFE films with micro-nano porosities. Given that the functional materials are sensitive in the vertical direction, we developed a structural design using the form of a multilayer stack to realize the independent multifunctional sensing.

An attractive feature of our devices is the multifunctional sensing functionality, especially for material identification. The devices exhibited a high pressure–sensing sensitivity of 15.22 kPa^−1^ and an accurate temperature resolution of 1 K based on the piezoresistance and thermoelectric property of the graphene/PDMS composite, respectively. Moreover, our devices were able to infer material properties based on the universal contact electrification. Notably, even with a simple contact-release movement, the devices can discriminate 10 common flat materials. The size of the devices could be further decreased using advanced responsive components, as summarized in fig. S7. Our devices exhibit prominent performances of pressure and temperature sensing by realizing a self-powered material identification (table S3). This is one of few works, to our knowledge, that use three mechanisms (piezoresistive, thermoelectric, and triboelectric effects) in a single device to achieve pressure, temperature, and material sensing.

For portable applications, sensing devices should be of low cost and power. We fabricated graphene/PDMS sponges via the template method due to its low cost and simple process with promising large-area production. To improve the performance of devices, we could develop microfluidic methods to create responsive components with a uniform distribution of pore size (*36*). On the basis of the sensing mechanism of the thermoelectric and the triboelectric effects, our devices exhibited self-powered performance, enabling their long-term monitoring applications with low consumption. One limitation of the device is the possible electron transfer of the electrification layer when it operates under the very hot or humid environment (*37*, *38*). The electron transfer will obstruct the accurate measurement of the output voltage via the electrification mechanism. An important research direction, therefore, might be the development of stable electrification layers and sensing mechanisms for special applications.

In summary, we have presented a simple, low-cost method to fabricate a multifunctional sensor using a hydrophobic PTFE film and a sponge-like graphene/PDMS composite. Using the piezoresistive and thermoelectric properties of the composite, our sensor exhibits a pressure sensitivity and self-powered temperature-sensing accuracy of 15.22 kPa^−1^ and 1 K, respectively. The use of contact electrification enables the material identification of objects. We hope that our methodologies offer an approach to multifunctional sensors with potential applications in wearable electronics and robotics.

## MATERIALS AND METHODS

### Materials

Graphene powder (P-11101) was purchased from Deyang Carbonene Technology Co. Ltd. PTFE preparation (60%), silver nitrate (AgNO_3_; 99.8%), and PVP (molecular weight, 1,300,000) were purchased from Shanghai Macklin Biochemical Technology Co. Ltd. Glycol (96%), sodium chloride (NaCl), zinc acetate (ZnAc_2_), and acetone (99.5%) were purchased from Bioroyee (Beijing) Biotechnology Co. Ltd. The commercial PDMS (SYLGARD 184) was purchased from Shanghai Smarttech Co. Ltd., where the silicone film was achieved by mixing part A and part B with a ratio of 1:10 in weight. All chemicals were used without further purification.

### Preparation of hydrophobic films

The schematic illustrations for the preparation procedure of hydrophobic films are shown in fig. S1A. In detail, 1 g of ZnAc_2_ and 1 g of NaCl were dissolved in 20 ml of deionized water and then mixed with 50 ml of PTFE emulsion. The mixed solution was stirred for 1 hour and cast into a flat template by blading method. The liquid films were dried in an oven at 100°C for 30 min, followed by curing at 400°C for 30 min. The resulting films were superhydrophilic. Using 1 M aqueous acetic acid, the NaCl and ZnO in the film were removed. After drying in an oven at 100°C for 30 min, the hydrophobic PTFE films were prepared. The thinnest hydrophobic PTFE films, produced by our method, were about 0.01 mm.

### Preparation of graphene/PDMS composites

The schematic illustrations for the preparation procedure are shown in fig. S1B. First, PDMS was mixed in elastomer and cross-linker with a mass of 2 and 0.2 g, and then a 0.055-g mass of graphene powder was added into the PDMS, flowed by mechanical stirring. Next, a 14-g mass of NaCl was mixed with the PDMS. Then, the mixture was poured into molds and compacted to designed samples via a tool. The samples were cured at 90°C for 60 min and then dissolved in hot water to remove NaCl particles. Last, the graphene/PDMS sponges were formed after drying in an oven at 90°C for 60 min.

### Synthesis of Ag NWs

Ag NWs were synthesized as described in the method (*39*). Typically, a 2.9-g mass of PVP was dissolved in a 254-g mass of glycol, forming solution A. A 3-g mass of AgNO_3_ was dissolved in a 9.9-g mass of glycol (solution B). Then, a 0.146-g mass of NaCl was dissolved in 10 ml of glycol (solution C). A solution and 480 μl of C solution were mixed in a three-necked flask in an oil bath at 90°C under mechanical stirring. Next, the B solution was slowly dissolved into the flask, flowed by mixing for 5 hours. When it was cooled down to room temperature, the resulting mixture was rinsed with acetone and distilled water.

### Preparation of the sensor

A 10-μl solution of prepared Ag NWs (0.3 mg ml^−1^) was dropped on the Cu sheets (10 mm by 10 mm) and then evaporated at room temperature (fig. S1C). Then, the fabricated graphene/PDMS composite (9 mm by 9 mm) was sandwiched between two Cu sheets. Last, the fabricated hydrophobic PTFE film (10 mm by 10 mm) was attached to the top Cu sheet via double-sided tape. First, the graphene/PDMS sponges were sandwiched between two Cu sheets. The UV-cured glue was dropped on the edges of the sponges. Then, the glue was cured via a UV lamp (fig. S1D).

### COMSOL simulation

The two-dimensional strain field and electric potential on the graphene/PDMS composite (Fig. 1F) were numerically calculated using the commercial software COMSOL. The width of the composite was 1 cm. The electric potential on the PTFE was numerically calculated, where the gap distance between the PTFE and object was 3 cm and the surface charge density was 0.02 μC m^−2^ in Fig. 1G.

### Algorithm simulation

The algorithm of material identification was calculated using the commercial software MATLAB. The database was developed by importing values via the function (importdata) and abstracting maximum value via the function (max). The distinguishing process used the function (find).

### Characterization and measurements

The morphology and crystalline structure of graphene/PDMS composites, hydrophobic PTFE films, and Ag NWs were investigated by a field-emission SEM (Hitachi, SU8020) and XRD with Cu Kα radiation (PANalytical X’ Pert3 Powder). Surface wetting of the prepared hydrophobic PTFE films was measured by a contact angle meter (SC1300F, China) at room temperature. A homemade system consisting of the single-column dynamometer (IMADA, MX2-500N) and dynamometer (IMADA, ZTA-50N) was used to supply force and measured the output press signals. A semiconductor thermoelectric cooler system was used to apply a different temperature. The output electrical signals of the device were measured by a digital source meter (Keithley, 2611B).

## Supplementary Material

abb9083_Movie_S3.mp4

abb9083_Movie_S1.mp4

abb9083_SM.pdf

abb9083_Movie_S2.mp4

## SUPPLEMENTARY MATERIALS

Supplementary material for this article is available at http://advances.sciencemag.org/cgi/content/full/6/34/eabb9083/DC1
