# Supplementary material for: Hierarchically patterned self-powered sensors for multifunctional tactile sensing
Source: Sci Adv. 2020 Aug 19;6(34):eabb9083. doi: 10.1126/sciadv.abb9083 (PMC7438107; doi:10.1126/sciadv.abb9083)
Supplement: abb9083_SM.pdf [file abb9083_SM.pdf]

Science Advances
AAAS

advances.sciencemag.org/cgi/content/full/6/34/eabb9083/DC1

Supplementary Materials for

Hierarchically patterned self-powered sensors for multifunctional tactile sensing

Yang Wang, Heting Wu, Lin Xu, Hainan Zhang, Ya Yang*, Zhong Lin Wang*

*Corresponding author. Email: yayang@binn.cas.cn (Y.Y.); zhong.wang@mse.gatech.edu (Z.L.W.)

Published 19 August 2020, *Sci. Adv.* **6**, eabb9083 (2020)
DOI: 10.1126/sciadv.abb9083

The PDF file includes:

Sections S1 to S4
Figs. S1 to S7
Tables S1 to S3
References

Other Supplementary Material for this manuscript includes the following:

(available at advances.sciencemag.org/cgi/content/full/6/34/eabb9083/DC1)

Movies S1 to S3

Section S1. Pressure sensing mechanism.

The sensing mechanism of the skin sensors is based on the piezoresistive properties of the graphene/ PDMS sponge. The resistance of the device can be divided to contact resistance between the electrodes and the graphene/ PDMS sponge and bulk resistance of the sponge. The resistance of the device is high with no pressure. With a small pressure, the hierarchical microstructures on the surface of the composite film will deform, leading to more conducting pathways between the electrodes and the graphene/ PDMS sponge. In our device, the AgNWs on the electrodes will enhance sensitivity in small pressure. The pores inside the sponge will be deformed, leading to reduce the resistance of the sponge. In this process, the sensitivity of the device is decided by the change of resistance of the sponge.

For the normal foams, the strain-pressure relationship in the low stain regime can be described as (*40*):

$$P = E\varepsilon \quad (\varepsilon < 0.5) \tag{1}$$

where P represents the applied pressure, E represents the elastic modulus of the graphene/ PDMS sponges, and ε represents the compressive strain (fig. S3E).

Section S2. Temperature sensing mechanism.

The temperature sensing of the sensor is due to the thermoelectric properties of the graphene/ PDMS sponge. The temperature of the stimuli (T_c) can be described as (*16*):

$$T_c = T_0 + \Delta T \tag{2}$$

$$\Delta T = \frac{V_{therm}}{S_T} \tag{3}$$

where V_{therm} is the output voltage, S_T is the Seebeck coefficient, ΔT is the temperature gradient, and T_0 is the constant temperature of the environment.

Section S3. The TENG mechanism.

Benefiting from universal contact electrification, any two different materials can induce triboelectric signals based on the coupling of contact electrification and electrostatic induction. When two materials contact each other, charge transfer appeared. According to the triboelectric series, electrons were injected from FEP films to the PTFE film. Due to form the balanced potential, no electron flow appeared in the external circuit in this process. When the FEP film was separated from the PTFE film by the external force, these triboelectric charges cannot be compensated. The resulting negative charges on the PTFE film can induce positive charges on the electrode, leading to free electrical flow from the electrode to the ground. The device exhibited output electrical signals in the detached process until the negative triboelectric charges on the PTFE are fully screened from the induced positive charges on the electrodes. When the FEP was approached to the PTFE film again, the electrons flow the ground to the electrode, leading to a reversed output signals. This process will keep until two films contact again (fig. S5).

Section S4. Success rate of material identification.

The process of material identification was operated with software MATLAB. The success rate (S) can be described as:

$$S = \frac{n}{N} \times 100 \tag{4}$$

where n is the number of successful identifications and N is the number of the test.

The electrical signals were measured by the sensor, and transformed to databases using MATLAB. For each object, we obtained twenty output independent electrical signals by using the sensor. Then signals were compared with the databases to infer their kinds (Table S1 and Table S2).

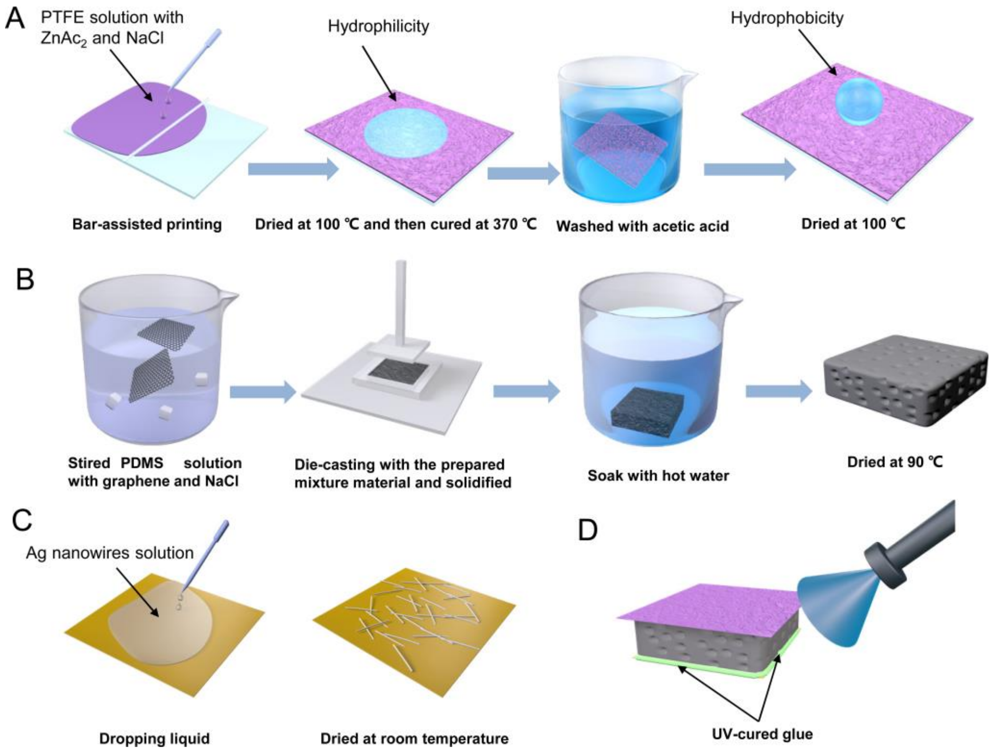

Fig. S1. Schematic of the whole fabrication process for the multifunctional sensor. (**A**) Preparation of superhydrophobic PTFE films. (**B**) Preparation of graphene/PDMS sponges. (**C**) Preparation of Cu sheet coating with Ag nanowires. (D) The assembly of the device.

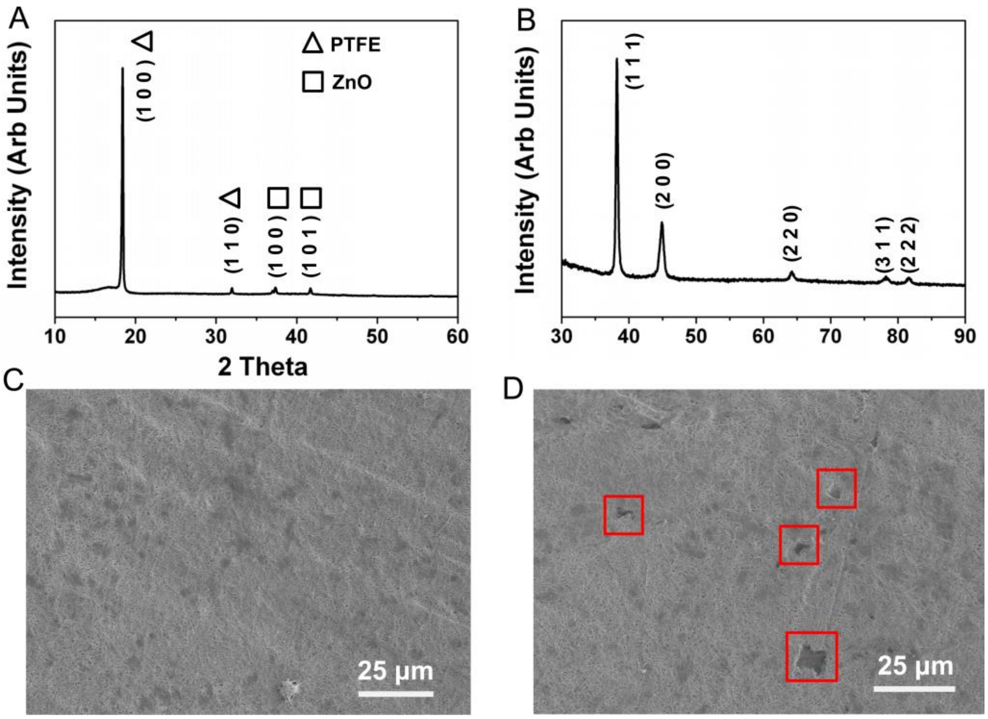

Fig. S2. The crystalline structure and SEM images of prepared materials. (**A**) XRD pattern of Zinc oxide filled PTFE composite. (**B**) XRD pattern of Ag NWs. (C) The SEM image of Ag NWs film. (D) The SEM image shows the film with the destroyed area (red square frame) after the stress test.

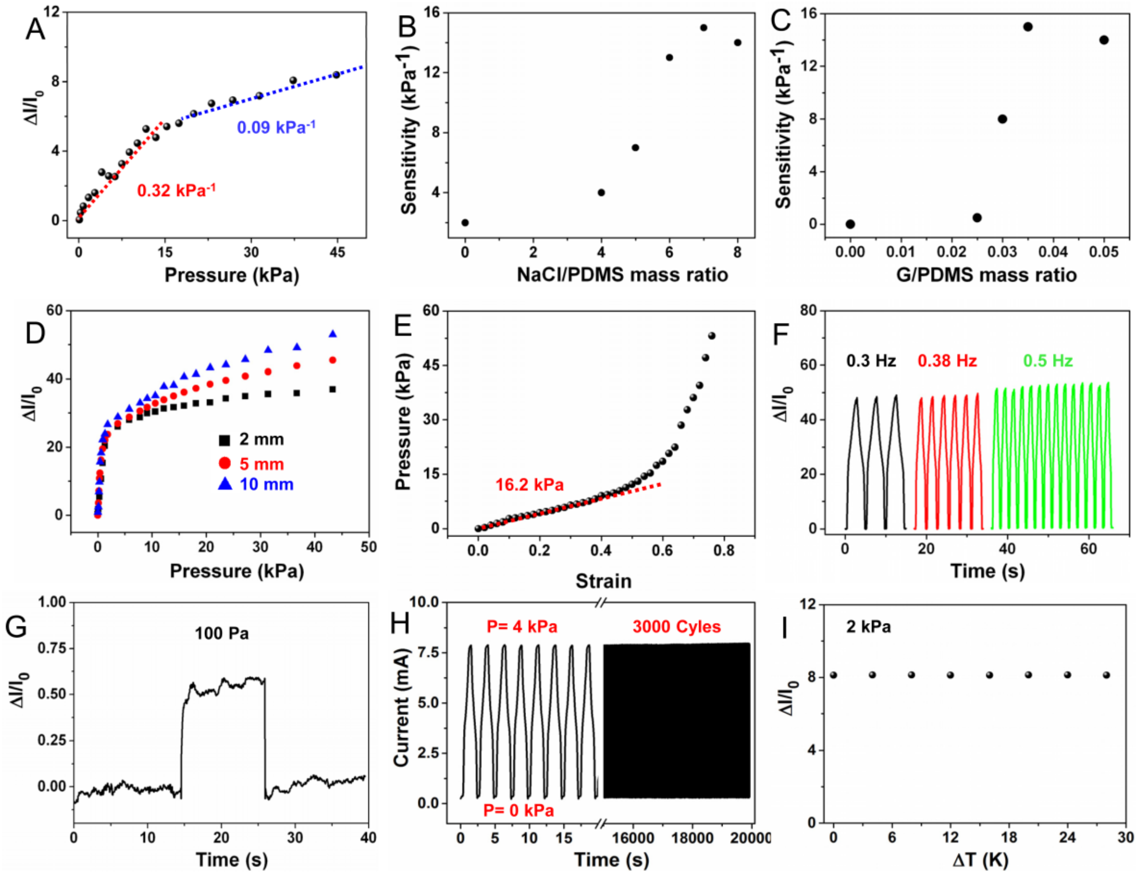

Fig. S3. The pressure responses of devices. (A) Sensitivity of the sensor without Ag NWs on the Cu sheet. (B) Sensitivity of the sensors with respect to NaCl/PDMS mass ratio. (C) Sensitivity of the sensors with respect to graphene/PDMS mass ratio. (D) Sensitivity of the sensors with respect to different thicknesses of the graphene/PDMS sponges. (E) Mechanical measurements of the graphene/PDMS sponge with increasing compressive strain. (F) Relative current change under different frequencies. (G) Current responses to loading/unloading the force of 0.01 N. (H) The durability test of a sensor under a pressure of 4 kPa. (I) Current change of devices as a function of temperature difference.

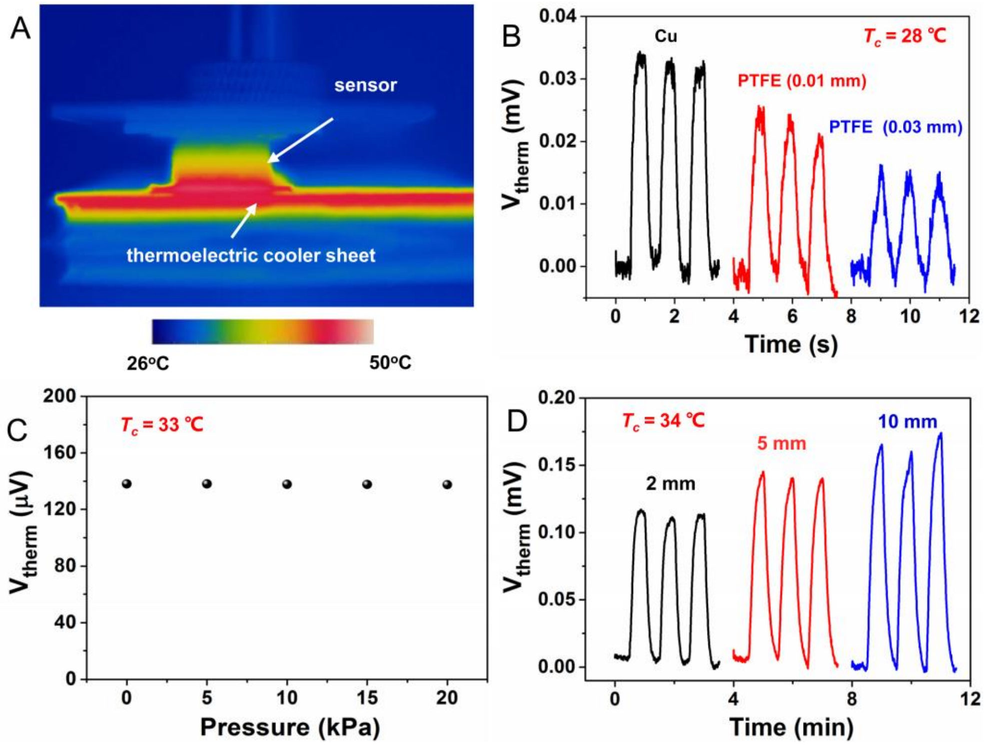

Fig. S4. The temperature responses of devices. (**A**) The home-made system containing a semiconductor thermoelectric cooler sheet as the heating source and the infrared thermal imaging to record temperature variations. (**B**) Measured thermal voltage corresponding to the Cu sheet, the PTFE film with 0.01 mm, and the PTFE film with 0.03 mm as the contact surface. T_C is the temperature of the object. (C) Measured thermal voltage as a function of pressure with a constant temperature. (D) Measured thermal voltage corresponding to different thicknesses of graphene/PDMS sponges.

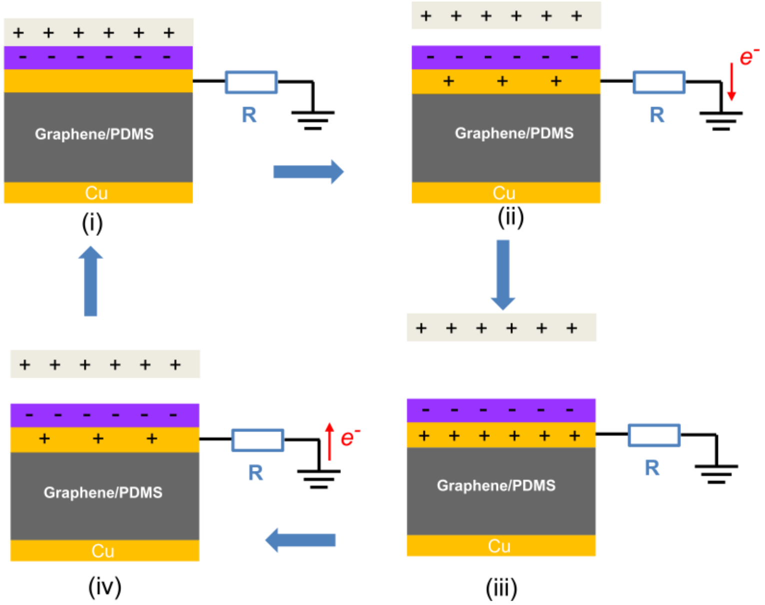

Fig. S5. The working principle of the TENG during one cycle.

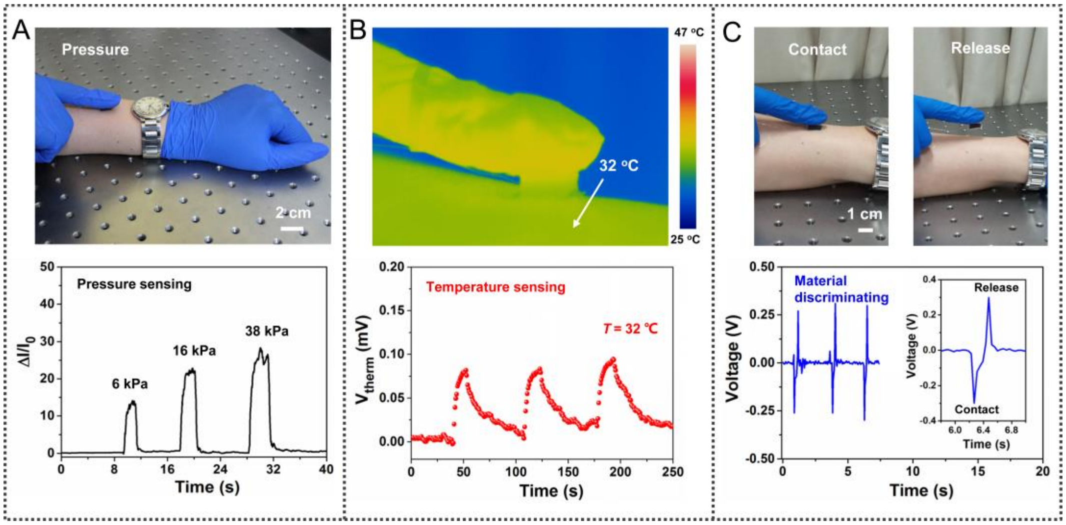

Fig. S6. Examples for the applications of sensors. (**A**) An optical image of a finger with the sensor touching human skin and a plot for the corresponding current change of the sensor. (**B**) An thermal image of a finger with the sensor touching human skin and a plot for the V_{therm} responses of the sensor. (**C**) Images showing operations of the finger to contact with the human skin and release. Plots showing contact-induced electrification responses to a contact-release movement. Photo Credit: Ya Yang, Beijing Institute of Nanoenergy and Nanosystems.

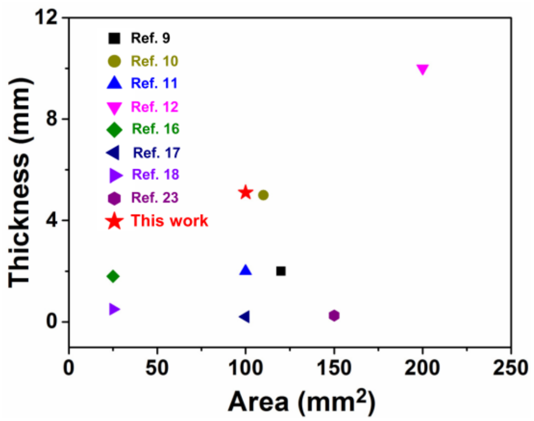

Fig. S7. Comparison of the size of our sensor with existing flexible sensors.

Table S1. The success rate of material identification for five objects.

Material	Success rate (%)
FEP	95
Acrylic	90
paper	90
Cu	80
Kapton	85

Table S2. The success rate of material identification for ten objects.

Material	Success rate (%)
FEP	80
Acrylic	70
paper	70
Cu	65
Kapton	70
Glass	60
Al	65
Wood	70
Cotton	70
Nylon	75

Table S3. The characteristics comparison of sensors based on various materials.

Materials	Pressure sensitivity	Pressure range	Temerature coefficient	Temerature range	Material identification	Other capabilities	References
PDMS /CNF	0.6 kPa^{-1}	0-140 kPa	×	×	×	×	**(9)**
Graphene /Polyurethane	0.26 kPa^{-1}	0-10 kPa	×	×	×	×	**(10)**
MXene	180.1	0-2 Strain	×	×	×	×	**(11)**
PDMS /CNF	0.031 kPa^{-1}	0-150 kPa	×	×	×	×	**(12)**
Organic thermoelectric	28.9 kPa^{-1} Self-powered	0-20 kPa	35.5 μV/K Self-powered	20-100 °C	×	×	**(16)**
rGO /PVDF	1 kPa^{-1}	0-25 kPa	2.93% K^{-1}	20-60 °C	×	×	**(17)**
SWNTs /PDMS	0.7 kPa^{-1}	0-25 kPa	0.83% K^{-1}	22-70 °C	×	×	**(18)**
Sensor array	×	×	×	×	Piezoresistive signals	×	**(23)**
Our work	15.22 kPa^{-1}	0-40 kPa	35.2 μV/K Self-powered	25-60 °C	Triboelectric signals Self-powered	Controling water drop	

REFERENCES AND NOTES

1. R. S. Johansson, J. R. Flanagan, Coding and use of tactile signals from the fingertips in object manipulation tasks. *Nat. Rev. Neurosci.* **10**, 345–359 (2009).

2. C. Bartolozzi, L. Natale, F. Nori, G. Metta, Robots with a sense of touch. *Nat. Mater.* **15**, 921–925 (2016).

3. D.-H. Kim, N. Lu, R. Ma, Y.-S. Kim, R.-H. Kim, S. Wang, J. Wu, S. M. Won, H. Tao, A. Islam, K. J. Yu, T.-i. Kim, R. Chowdhury, M. Ying, L. Xu, M. Li, H.-J. Chung, H. Keum, M. M. Cormick, P. Liu, Y.-W. Zhang, F. G. Omenetto, Y. Huang, T. Coleman, J. A. Rogers, Epidermal electronics. *Science* **333**, 838–843 (2011).

4. S. Xu, Y. Zhang, L. Jia, K. E. Mathewson, K.-I. Jang, J. Kim, H. Fu, X. Huang, P. Chava, R. Wang, S. Bhole, L. Wang, Y. J. Na, Y. Guan, M. Flavin, Z. Han, Y. Huang, J. A. Rogers, Soft microfluidic assemblies of sensors, circuits, and radios for the skin. *Science* **344**, 70–74 (2014).

5. Y. H. Jung, B. Park, J. U. Kim, T.-I. Kim, Bioinspired electronics for artificial sensory systems. *Adv. Mater.* **31**, e1803637 (2019).

6. Z. Xie, R. Avila, Y. Huang, J. A. Rogers, Flexible and stretchable antennas for biointegrated electronics. *Adv. Mater.* **32**, 1902767 (2020).

7. A. Chortos, J. Liu, Z. Bao, Pursuing prosthetic electronic skin. *Nat. Mater.* **15**, 937–950 (2016).

8. A. Miyamoto, S. Lee, N. F. Cooray, S. Lee, M. Mori, N. Matsuhisa, H. Jin, L. Yoda, T. Yokota, A. Itoh, M. Sekino, H. Kawasaki, T. Ebihara, M. Amagai, T. Someya, Inflammation-free, gas-permeable, lightweight, stretchable on-skin electronics with nanomeshes. *Nat. Nanotechnol.* **12**, 907–913 (2017).

9. S. Wu, J. Zhang, R. B. Ladani, A. R. Ravindran, A. P. Mouritz, A. J. Kinloch, C. H. Wang, Novel electrically conductive porous PDMS/carbon nanofiber composites for deformable strain sensors and conductors. *ACS Appl. Mater. Interfaces* **9**, 14207–14215 (2017).

10. H.-B. Yao, J. Ge, C.-F. Wang, X. Wang, W. Hu, Z.-J. Zheng, Y. Ni, S.-H. Yu, A flexible and highly pressure-sensitive graphene-polyurethane sponge based on fractured microstructure design. *Adv. Mater.* **25**, 6692–6698 (2013).

11. Y. Ma, N. Liu, L. Li, X. Hu, Z. Zou, J. Wang, S. Luo, Y. Gao, A highly flexible and sensitive piezoresistive sensor based on MXene with greatly changed interlayer distances. *Nat. Commun.* **8**, 1207 (2017).

12. Y. Song, H. Chen, Z. Su, X. Chen, L. Miao, J. Zhang, X. Cheng, H. Zhang, Highly compressible integrated supercapacitor-piezoresistance-sensor system with CNT-PDMS sponge for health monitoring. *Small* **13**, 1702091 (2017).

13. A. Nag, S. C. Mukhopadhyay, J. Kosel, Wearable flexible sensors: A review. *IEEE Sens. J.* **17**, 3949–3960 (2017).

14. C. Yan, J. Wang, P. S. Lee, Stretchable graphene thermistor with tunable thermal index. *ACS Nano* **9**, 2130–2137 (2015).

15. T. Q. Trung, S. Ramasundaram, B.-U. Hwang, N.-E. Lee, An all-elastomeric transparent and stretchable temperature sensor for body-attachable wearable electronics. *Adv. Mater.* **28**, 502–509 (2016).

16. F. Zhang, Y. Zang, D. Huang, C.-a. Di, D. Zhu, Flexible and self-powered temperature-pressure dual-parameter sensors using microstructure-frame-supported organic thermoelectric materials. *Nat. Commun.* **6**, 8356 (2015).

17. J. Park, M. Kim, Y. Lee, H. S. Lee, H. Ko, Fingertip skin-inspired microstructured ferroelectric skins discriminate static/dynamic pressure and temperature stimuli. *Sci. Adv.* **1**, e1500661 (2015).

18. G. Y. Bae, J. T. Han, G. Lee, S. Lee, S. W. Kim, S. Park, J. Kwon, S. Jung, K. Cho, Pressure/temperature sensing bimodal electronic skin with stimulus discriminability and linear sensitivity. *Adv. Mater.* **30**, e1803388 (2018).

19. B. Belzile, L. Birglen, Stiffness analysis of underactuated fingers and its application to proprioceptive tactile sensing. *IEEE ASME Trans. Mechatron.* **21**, 2672–2681 (2016).

20. Z. Kappassov, J.-A. Corrales, V. Perdereau, Tactile sensing in dexterous robot hands. *Robot. Auton. Syst.* **74**, 195–220 (2015).

21. H. Liu, F. Sun, Material identification using tactile perception: A semantics-regularized dictionary learning method. *IEEE ASME Trans. Mechatron.* **23**, 1050–1058 (2018).

22. G. Zhai, J. Chen, S. Wang, K. Li, L. Zhang, Material identification of loose particles in sealed electronic devices using PCA and SVM. *Neurocomputing* **148**, 222–228 (2015).

23. S. Sundaram, P. Kellnhofer, Y. Li, J.-Y. Zhu, A. Torralba, W. Matusik, Learning the signatures of the human grasp using a scalable tactile glove. *Nature* **569**, 698–702 (2019).

24. X. Pu, M. Liu, X. Chen, J. Sun, C. Du, Y. Zhang, J. Zhai, W. Hu, Z. L. Wang, Ultrastretchable, transparent triboelectric nanogenerator as electronic skin for biomechanical energy harvesting and tactile sensing. *Sci. Adv.* **3**, e1700015 (2017).

25. Z. L. Wang, J. Chen, L. Lin, Progress in triboelectric nanogenerators as a new energy technology and self-powered sensors. *Energ. Environ. Sci.* **8**, 2250–2282 (2015).

26. S. Chun, C. Pang, S. B. Cho, A micropillar-assisted versatile strategy for highly sensitive and efficient triboelectric energy generation under in-plane stimuli. *Adv. Mater.* **32**, 1905539 (2020).

27. M. Ha, S. Lim, S. Cho, Y. Lee, S. Na, C. Baig, H. Ko, Skin-inspired hierarchical polymer architectures with gradient stiffness for spacer-free, ultrathin, and highly sensitive triboelectric sensors. *ACS Nano* **12**, 3964–3974 (2018).

28. M. Shi, J. Zhang, H. Chen, M. Han, S. A. Shankaregowda, Z. Su, B. Meng, X. Cheng, H. Zhang, Self-powered analogue smart skin. *ACS Nano* **10**, 4083–4091 (2016).

29. R. Hinchet, H.-J. Yoon, H. Ryu, M.-K. Kim, E.-K. Choi, D.-S. Kim, S.-W. Kim, Transcutaneous ultrasound energy harvesting using capacitive triboelectric technology. *Science* **365**, 491–494 (2019).

30. H. Guo, X. Pu, J. Chen, Y. Meng, M.-H. Yeh, G. Liu, Q. Tang, B. Chen, D. Liu, S. Qi, C. Wu, C. Hu, J. Wang, Z. L. Wang, A highly sensitive, self-powered triboelectric auditory sensor for social robotics and hearing aids. *Sci. Robot.* **3**, eaat2516 (2018).

31. J. Liang, L. Li, D. Chen, T. Hajagos, Z. Ren, S.-Y. Chou, W. Hu, Q. Pei, Intrinsically stretchable and transparent thin-film transistors based on printable silver nanowires, carbon nanotubes and an elastomeric dielectric. *Nat. Commun.* **6**, 7647 (2015).

32. H. Zou, Y. Zhang, L. Guo, P. Wang, X. He, G. Dai, H. Zheng, C. Chen, A. C. Wang, C. Xu, Z. L. Wang, Quantifying the triboelectric series. *Nat. Commun.* **10**, 1427 (2019).

33. S. Lin, L. Xu, L. Zhu, X. Chen, Z. L. Wang, Electron transfer in nanoscale contact electrification: Photon excitation effect. *Adv. Mater.* **31**, e1901418 (2019).

34. T. Xu, L.-P. Xu, X. Zhang, S. Wang, Bioinspired superwettable micropatterns for biosensing. *Chem. Soc. Rev.* **48**, 3153–3165 (2019).

35. Y. Zhao, C. Yu, H. Lan, M. Cao, L. Jiang, Improved interfacial floatability of superhydrophobic/superhydrophilic Janus sheet inspired by lotus leaf. *Adv. Funct. Mater.* **27**, 1701466 (2017).

36. P. Zhu, T. Kong, X. Tang, L. Wang, Well-defined porous membranes for robust omniphobic surfaces via microfluidic emulsion templating. *Nat. Commun.* **8**, 15823 (2017).

37. C. X. Lu, C. B. Han, G. Q. Gu, J. Chen, Z. W. Yang, T. Jiang, C. He, Z. L. Wang, Temperature effect on performance of triboelectric nanogenerator. *Adv. Eng. Mater.* **19**, 1700275 (2017).

38. Y. Hu, X. Wang, H. Li, H. Li, Z. Li, Effect of humidity on tribological properties and electrification performance of sliding-mode triboelectric nanogenerator. *Nano Energy* **71**, 104640 (2020).

39. J.-Y. Lin, Y.-L. Hsueh, J.-J. Huang, The concentration effect of capping agent for synthesis of silver nanowire by using the polyol method. *J. Solid State Chem.* **214**, 2–6 (2014).

40. L. Gong, S. Kyriakides, W.-Y. Jang, Compressive response of open-cell foams. Part I: Morphology and elastic properties. *Int. J. Solids. Struct.* **42**, 1355–1379 (2005).
